# Diagnostic significance of dysregulated miRNAs in T-cell malignancies and their metabolic roles

**DOI:** 10.3389/fonc.2023.1230273

**Published:** 2023-08-10

**Authors:** Deepankar Mondal, Sapnita Shinde, Souvik Paul, Suresh Thakur, GSK Velu, Atul Kumar Tiwari, Vineeta Dixit, Ajay Amit, Naveen Kumar Vishvakarma, Dhananjay Shukla

**Affiliations:** ^1^ Department of Biotechnology, Guru Ghasidas Vishwavidyalaya, Bilaspur, Chhattisgarh, India; ^2^ Department of Surgical Gastroenterology, All India Institute of Medical Sciences, Raipur, Chhattisgarh, India; ^3^ Centre for Excellence in Genomics, Trivitron Healthcare Pvt. Ltd., Chennai, India; ^4^ Department of Zoology, Dr. Bhawan Singh Porte Government College, Pendra, Chhattisgarh, India; ^5^ Department of Botany, Sri Satguru Jagjit Singh Namdhari College, Gharwa, Jharkhand, India; ^6^ Department of Forensic Sciences, Guru Ghasidas Vishwavidyalaya, Bilaspur, Chhattisgarh, India

**Keywords:** T-cell malignancies, MicroRNAs, diagnosis, lymphomas, prognosis, metabolism

## Abstract

T-cell malignancy is a broad term used for a diverse group of disease subtypes representing dysfunctional malignant T cells transformed at various stages of their clonal evolution. Despite having similar clinical manifestations, these disease groups have different disease progressions and diagnostic parameters. The effective diagnosis and prognosis of such a diverse disease group demands testing of molecular entities that capture footprints of the disease physiology in its entirety. MicroRNAs (miRNAs) are a group of noncoding RNA molecules that regulate the expression of genes and, while doing so, leave behind specific miRNA signatures corresponding to cellular expression status in an altered stage of a disease. Using miRNAs as a diagnostic tool is justified, as they can effectively distinguish expressional diversity between various tumors and within subtypes of T-cell malignancies. As global attention for cancer diagnosis shifts toward liquid biopsy, diagnosis using miRNAs is more relevant in blood cancers than in solid tumors. We also lay forward the diagnostic significance of miRNAs that are indicative of subtype, progression, severity, therapy response, and relapse. This review discusses the potential use and the role of miRNAs, miRNA signatures, or classifiers in the diagnosis of major groups of T-cell malignancies like T-cell acute lymphoblastic lymphoma (T-ALL), peripheral T-cell lymphoma (PTCL), extranodal NK/T-cell lymphoma (ENKTCL), and cutaneous T-cell lymphoma (CTCL). The review also briefly discusses major diagnostic miRNAs having prominent metabolic roles in these malignancies to highlight their importance among other dysregulated miRNAs.

## 1 Introduction

Cancer research has made remarkable progress in recent years, bringing many potent drugs to the fore for its treatment and management. However, the effectiveness of these cancer therapies largely depends on accurate and timely diagnosis. In most cases, delayed or inaccurate diagnosis leads to faulty clinical judgment, which incurs toxic responses in patients and renders therapeutic endeavors less effective (1). This disadvantage of diagnostic delays and inaccuracy can be overcome by investigating circulating fluids that may be home to disease-specific markers, especially in cases of lymphoma more than in solid tumors (2). Although the incidence of various T-cell lymphomas may be statistically rare, it is considered very aggressive and is often associated with poorer disease outcomes (3). Patients harboring such malignancies can also face treatment failures, as they develop resistance to established chemotherapeutic regimens faster than solid tumors (4). To diagnose such aggressive malignancies, conventional methods may not be sufficient to identify these diseases as early as possible. However, the growing prospects of liquid biopsy can be exploited to diagnose these lymphomas (5). One such biomarker is microRNAs (miRNAs), whose global expression can be investigated not only in patient tissues but also in their circulating fluids, providing scope for liquid biopsy (6). However, miRNA profiling, as a diagnostic option is still limited within the research domain; its applicability in routine clinical practice has not yet materialized as it requires high-throughput techniques. The present review describes the diagnostic and prognostic potential of miRNAs that are relevant for the efficient discriminatory diagnosis of a particular T-cell lymphoma from a wide spectrum of T-cell malignant lymphomas (TCLs).

miRNAs were first discovered in *Caenorhabditis elegans* as *lin-4*, a noncoding RNA reported to downregulate the expression of another gene, *lin-14* (7). miRNAs are short (usually 21–23 nucleotides), noncoding (nc), ssRNA molecules produced endogenously in animal and plant cells that function posttranscriptionally, consequently regulating gene expression. Most often, miRNAs repress gene expression by binding to the 3′untranslated regions (3′UTRs) of their respective mRNA targets, thus cleaving them to suppress translation (8). These miRNAs can co-express and co-regulate one or more cellular pathways and result in a phenotypic consequence of oncogenic or onco-suppressive functions. miRNAs and their repressive roles have been widely studied using both *in vitro* and *in silico* models, in addition to their gene-inducing capabilities (7–9).

**Figure 1:**
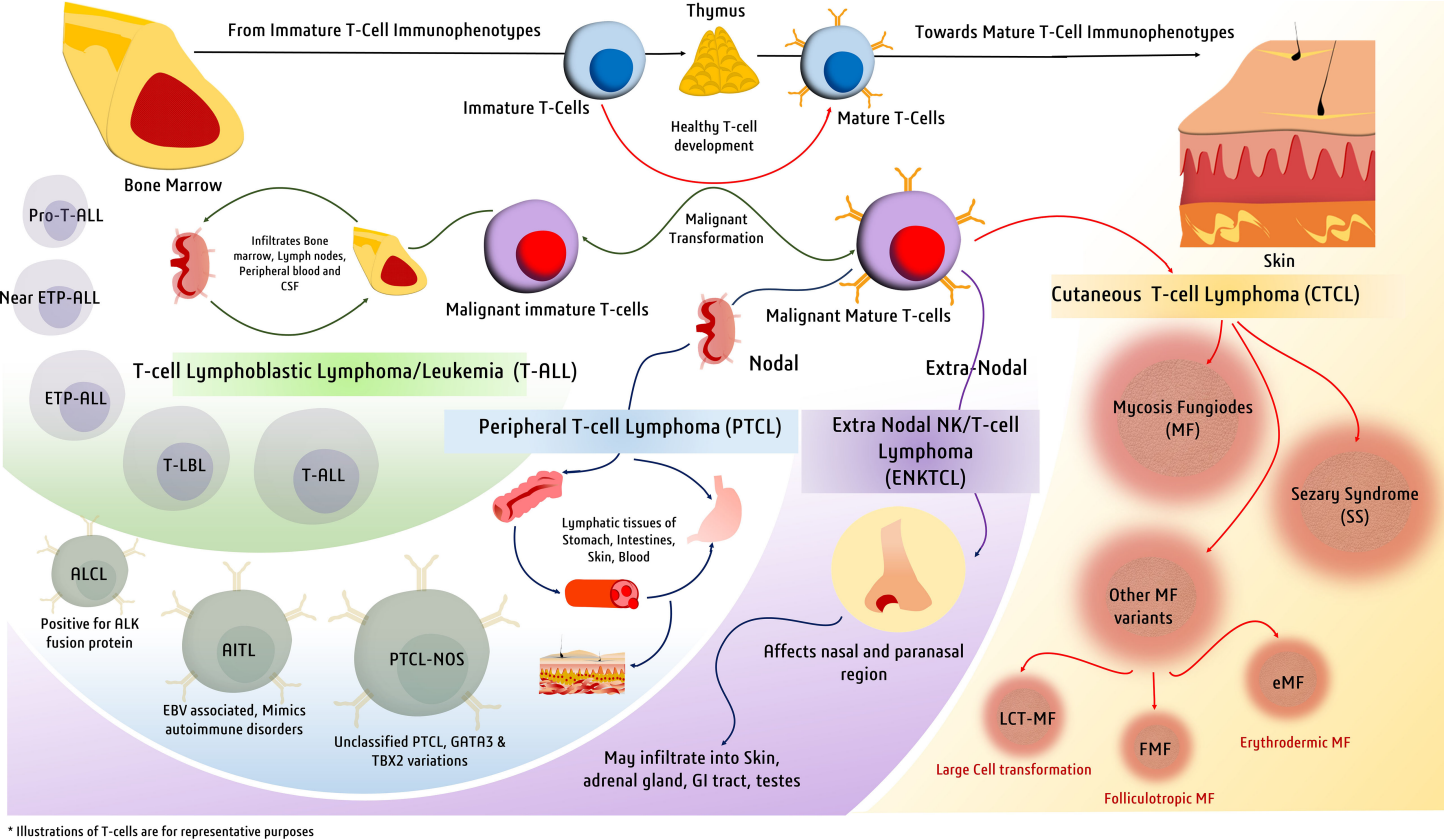
Major types of T-cell malignancies. Malignant transformation at various stages of T-cell development gives rise to a spectrum of malignancies defined by a variety of clinical manifestations.

Cancers have overwhelming involvement in multiple pathways, for which miRNAs certainly create a miRNA signature while regulating those pathways and may indicate the cell’s physiological, metabolic, pathologic, and tumor-progression status (10). It is also noteworthy that T-cell malignant formations shed circulating miRNAs in circulating body fluids, which can be quantified to design promising diagnostic applications and monitor therapy response during treatment (10). In this review, we discuss different miRNAs with important diagnostic and prognostic applications in various types of T-cell malignancies. For this review, the diversity of T-cell malignancies is based on various pathological studies and the WHO classification (11). However, a clear classification is yet to be explored and is beyond the scope of this review (12). Hence, we discuss major T-cell malignancies and their dysregulated miRNAs, contributing to aspects of diagnostic or prognostic values in adult T-lymphoblastic leukemia/lymphoma (T-ALL), peripheral T-cell lymphoma (PTCL) and its types, extranodal T-cell lymphoma (ENKTCL), and cutaneous T-cell lymphoma (CTCL) (**Figure 1**). We also discuss how diagnostic miRNAs have a significant role in reprogramming cancer metabolism, as we underline emerging evidence suggesting a crucial role of miRNAs in regulating metabolic processes such as insulin sensitivity, glucose and lipid metabolism, and energy homeostasis. As metabolic miRNAs are being used for diagnostic and prognostic applications in metabolic disorders (13), they can have similar diagnostic and prognostic utilities in cancers as well, which undergo metabolic reprogramming as a key hallmark toward transformation. A recent computational biology study using machine learning has identified seven key miRNAs associated with mRNAs involved in lipid metabolism that are differentially expressed between prostate cancer and its benign counterpart. The differential expression of this lipid metabolism-related signature was also validated by PCR and in publicly available datasets (14). In addition to forming a molecular signature, miRNA expression profiles can identify distinct metabolic phenotypes within a disease spectrum. By identifying patients who may respond differently to a particular therapy or need specialized care, sub-phenotyping patients based on miRNA signatures can help precision medicine efforts (15, 16). Therefore, miRNAs along with their downstream metabolite signatures, can also potentially distinguish high-risk patient groups from others (17). The diagnostic and prognostic potential of miRNA profiling can be harnessed in clinical settings if it can be narrowed down to a few miRNAs defining a particular cancer subtype. The diagnostic focus required to distinguish a spectrum of disease manifestations can also be achieved by quantifying key miRNAs and their metabolic targets. Moreover, finding out how dysregulated miRNAs affect functional outcomes can reveal their role in metabolic processes verified through *in vitro* and *in vivo* research, which can also prove the miRNAs’ potential as therapeutic targets.

## 2 T-lymphoblastic leukemia/lymphoma

According to the International Lymphoma study group and the WHO, T-lymphoplastic leukemia/lymphoma comprises two kinds of malignancies namely T-cell lymphoblastic lymphoma (T-LBL) and T-ALL (18, 19). Both are malignancies of immature thymocytes that share large similarities in morphology and immunophenotypes (20, 21). Both malignancies have considerable infiltration in the mediastinum, differing in their proportion of bone marrow infiltration. T-ALL has more (>25%) bone marrow infiltration, whereas T-LBL has considerably high infiltration in the lymph nodes along with low infiltration in the bone marrow (<25%). Malignant thymocytes in T-LBL and T-ALL also infiltrate peripheral blood, bone marrow, and cerebrospinal fluid (22). As far as genetic and epigenetic aberrations in both T-LBL and T-ALL are concerned, their molecular features largely overlap with some differential mutations in each. The early T-cell precursor LBL/ALL (ETP-LBL or ETP-ALL) is another aggressive subtype of immature T-cell malignancy whose mutational and transcriptional profiles closely resemble those of myeloid progenitors and hematopoietic stem cells (21, 22). However, there exist other immunophenotypic subgroups, such as Near-ETP-ALL and Pro-T-ALL, whose distinct phenotypic and clinical presentations are not clear enough (23).

These T-ALL malignancies can be sub-grouped based on the distinct expression of various transcription factors, such as TAL1/TAL2, TLX1/TLX3, HOXA, and LMO1/LYL1, collectively known as type-A abnormalities (24). T-ALL is reported to have dysregulation of cell cycle regulation pathways such as the NOTCH-MYC-FBXW7 pathway, CDKN2A, CDK4/6 complex, kinase pathways such as JAK/STAT5 and PIK3/AKT/MTOR, and RNA metabolism pathways such as RPL5/10/11/22, CNOT3, and EIF4A (25). MicroRNAs involved in regulating these pathways are expected to have preferential diagnostic potential. In the case of T-ALL, miRNAs regulating the NOTCH1 pathway to achieve unchecked proliferation, apoptosis inhibition, and resistance to drug therapies may play a significant role in diagnosis (25). MicroRNAs, such as miR-128-3p, 148a-3p, miR-181a/b, miR-363-3p, and miR-20b-5p, are also involved in the pathogenesis of T-ALL (26). Other miRNAs suspected to regulate T-ALL pathobiology are miR-342, miR-223, miR-150, miR-142-3p, miR-93, miR-92, miR-26a, miR-20a, miR-19b, and miR-16 (27) (**Table 1**).

**Table 1:** MicroRNAs influencing notable clinical outcomes in T-ALL.

miRNA	Status	Significance	Metabolic significance	Reference
miR-16	Overexpression	Higher survival	Reprogram glycolysis and chemoresistance	Xi et al. (28) and Zhao et al. (29)
miR-17-5p	Under-expression	Poor prognosis	Regulates glycolytic and oxidative metabolism	Akbari Moqadam et al. (30) and Izreig et al. (31)
miR-99a, miR-125b	OE	Poor survival, express more in the TLK3 T-ALL subtype	Glycolytic downregulation	Hui et al. (32) Liu et al. (33) Peng et al. (34) Renou et al. (35), and Wang et al. (36)
miR-100	OE	Lower survival, poor disease-free survival, vincristine resistance	Reduced lipid accumulation and inositol phosphate metabolism	Hassan et al. (37) and Wei et al. (38)
miR-124a	UE	Poor prognosis, shorter survival, vincristine resistance	Inhibits Warburg effect	Agirre et al. (39) and Sun et al. (40)
miR-128b	UE	Poor prognosis, poor response to prednisone	Inhibits glucose metabolism, mitochondrial respiration	Nemes et al. (41) and Xiao et al. (42)
miR-128-3p	UE	Poor prognosis	Inhibits glucose metabolism, mitochondrial respiration	Akbari Moqadam et al. (30) and Xiao et al. (42)
miR-142-3p	OE	Increased sensitivity to steroid therapy	Increased oxygen, glucose, and lactate production	Correia and Barata (27) and Liu et al. (43)
miR-221	OE	Lowest 5-year survival; express more in ETP-ALL	Related to adipogenesis	Coskun et al. (44) and Yamaguchi et al. (45)
miR-222	OE	Poor response to induction therapy, poor prognosis	Related to adipogenesis	Coskun et al. (44) and Yamaguchi et al. (45)
miR-326	UE	Poor prognosis, shorter survival, resistance to therapies	Glucose metabolism	Ghodousi et al. (46) and Hatziapostolou et al. (47)
miR-708	UE	Poor response to prednisone, higher risk of relapse, associated with childhood T-ALL	Facilitates cellular metabolism	Monteleone and Lutz (48) and Rodríguez-Comas et al. (49)

### 2.1 miRNAs having diagnostic significance in T-ALL

**Figure 2:**
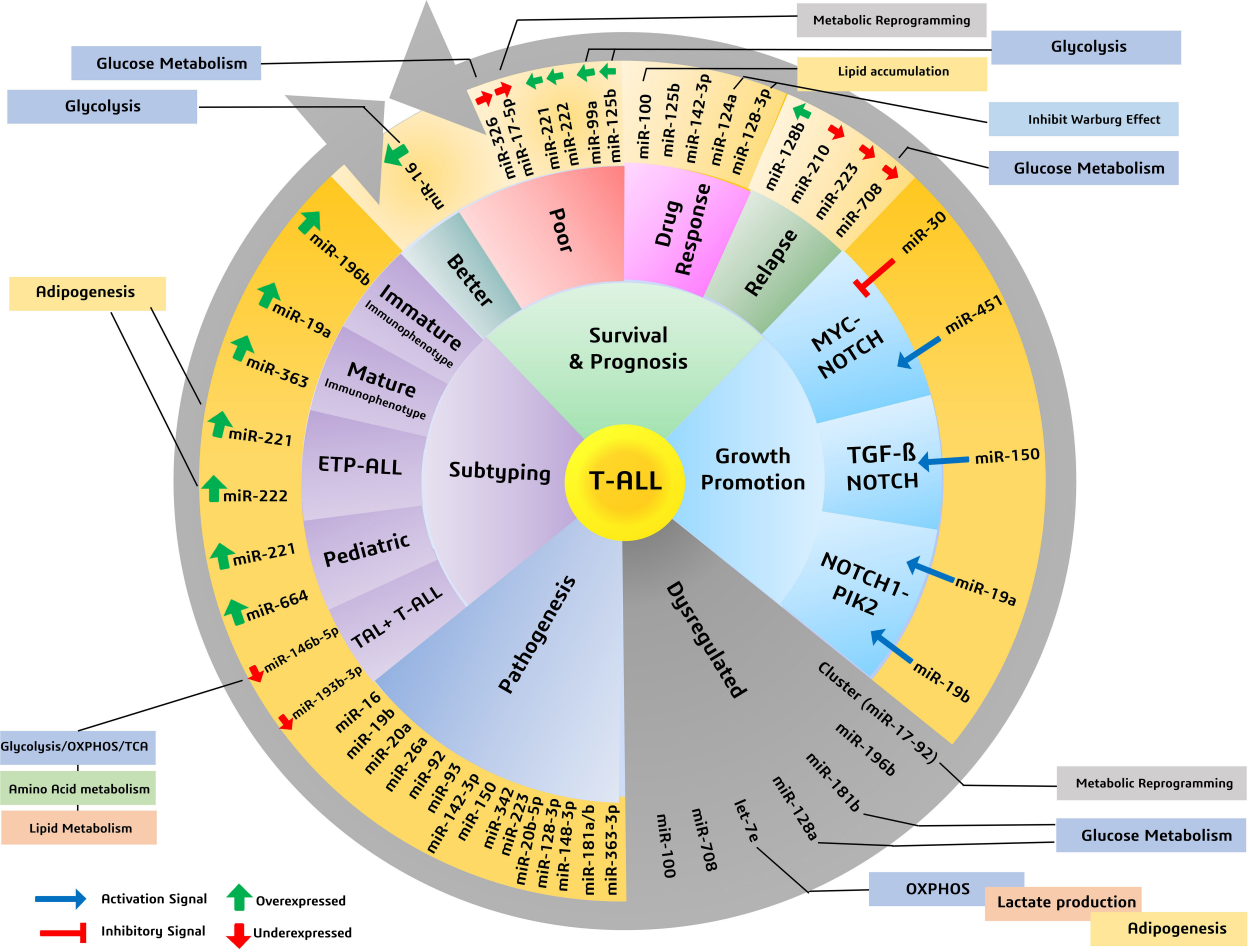
Various miRNAs and their significance in patient survival outcomes, prognosis, and subtyping, highlighting miRNAs involved in metabolic reprogramming of cancers.

When T-ALL microRNA expression was compared with that of healthy T cells, higher expression of miR-128a and miR-181b was observed alongside significant underexpression of miR-100 and let-7e (50). A study noted a general downregulation of miR-708 among all T-cell malignant patient groups, whereas miR-196b was found to be upregulated significantly only in patients with T-ALL immunophenotypes (51). A separate study evaluated the increased expression of seven miRNAs (miR-92, miR-20a, miR-19a, miR-19b, miR-18a, miR-17-3p, and miR-17-5p) in the miRNA cluster (miR-17-92) and reported their involvement in chromosomal rearrangement activity, including translocation (27). Among these miRNAs, significant overexpression of miR-19b and miR-19a has been reported in patients with T-ALL. An miRNA panel that co-expresses miR-26a and miR-92, along with miR-19a and miR-19b, is strongly associated with T-ALL patients (52). Moreover, among miRNA species that are underexpressed, miR-150 has a significant role in increasing hematopoietic progenitor growth and cell proliferation in T-ALL (53). The underexpressing miRNAs, miR-30 and miR-451, are believed to be tumor suppressive in nature, inhibiting NOTCH1 and NOTCH2 expression. These aforementioned miRNAs play a significant role in regulating the signaling pathways involved in the progression of T-ALL (**Figure 2**) (54).

In a study, several miRNAs were reported to have stage- and subtype-specific expression statuses. For instance, upregulated miR-196b in T-ALL corresponds with a more immature immunophenotype of malignant T cells, whereas upregulated miR-363 and miR-19a correspond with a more mature malignant T-cell immunophenotype (44, 55). Another miRNA couple, miR-221 and miR-222, have high expression in T-ALL cases but more significantly in its ETP-ALL subtype, as high as eight- and fivefold, respectively (56). Moreover, an independent study used microRNA profiling and noted the under-expression of miR-124a, miR-146b-5p, miR-150, miR-193b-3p, miR-326, miR-451, miR-30, and miR-708 in T-ALL. Among them, lower expression of miR-146b-5p was correlated with the TAL1+ T-ALL patient group (35). Lower expression of a different miRNA, miR-193b-3p, was associated with the TAL-rearranged T-ALL subtype and the NOTCH-induced T-ALL subgroup. MicroRNAs can also differentially associate with adult and pediatric T-ALL disease biology; for instance, in children with T-ALL, miR-221 was found to overexpress significantly in their peripheral blood mononuclear cells (PBMNCs). Interestingly, miR-221 is also associated with the refractory stage of T-ALL, signifying its prognostic role in both adult and pediatric cases. Similarly, a different miRNA (miR-664) was found to be overexpressed in pediatric T-ALL, underlying a possible association with miR-221 (**Figure 2**). Additionally, miR-664 has been reported to confer malignant features such as cell growth promotion, cell migration, and apoptotic inhibition of cells in T-ALL (51) (**Table 1**).

### 2.2 miRNAs and prognosis of T-ALL disease

Dysregulated miRNAs can also indicate survival outcomes in patients; for instance, a miRNA signature of overexpressed miR-221 and miR-222 in T-ALL, more so in ETP-ALL, is reported to be associated with the lowest 5-year survival scores and is also known to induce chemoresistance (44). Overexpression of miR-221 was found to be associated with poorer response to induction therapy, risk typing, and blood cell counts, making it a reliable marker for the diagnosis and prognosis of T-ALL (57). A different pair of miR-99a and miR-125b is associated with the TLK3 T-ALL subtype that exhibits poorer patient survival (35). Patients diagnosed with T-ALL have lower expression of miR-30 and miR-100, and while the role of miR-30 is still under investigation, miR-100 underexpression correlates with poor overall and disease-free survival rates, making it a high-risk prognostic biomarker (27, 37). As high expression of miR-30 in breast cancer is associated with better survival and prognosis (58), its low expression in T-ALL may possibly resemble poor survival, which requires further investigation. Lower expression of miR-326, along with high expression of BAALC (brain and acute lymphoblastic leukemia cytoplasmic protein), is associated with poor survival, especially in children, along with leukemogenesis, shorter survival, resistance to therapy, and MRD (minimal residual disease) (46). There are other miRNAs, for instance, miR-16, which, despite having a nonsignificant overexpression in T-ALL, gave better 1-year survival rates as high as 50% in patients having comparatively higher levels of miR-16 (28). Another, miR-142-3p, is overexpressed in T-ALL cells and is involved in the downregulation of the cAMP/PKA pathway, causing enhanced cell migration. On the one hand, this miRNA causes increased sensitivity to steroid therapy; on the other hand, its inhibition causes increased sensitivity to dexamethasone in patients with prednisone resistance (27). A poorer response to prednisone is also associated with lower expression of miR-708, a miRNA associated with childhood T-ALL. Higher levels of miR-708 can be of diagnostic significance and can signify moderate survival, whereas lower expression of miR-708 is associated with higher risk and higher chances of relapse (59) (**Figure 2**) (**Table 1**).

### 2.3 Metabolic roles of dysregulated miRNAs in T-ALL

T-ALL finds significant overexpression and underexpression of many miRNAs, amongst which we discuss prominently dysregulated miRNAs having metabolic roles, either reported in T-cell malignancies or in other cancers. In T-ALL, an upregulated miR-16, positively influencing patient survival (28), is reported to reprogram glycolysis and chemoresistance (29) in cervical cancer by activating PKD4, and also increase the radiosensitivity of prostate cancer cells (60). A downregulated let-7e in T-ALL was reported to regulate metabolic rewiring by inhibiting oxidative phosphorylation and adipogenesis and increasing lactate accumulation in a breast cancer study (61). A negative prognostic factor of underexpressing miR-17-5p in T-ALL leads to tumor metabolic reprogramming *via* its cluster miR-17-92 in B-lymphoma cells (31). Similarly, miR-99a and miR-125b, resembling poor prognosis and patient survival, were reported to be involved in glycolytic downregulation (34, 36) by facilitating decreased glucose uptake and oxygen consumption (32, 33). Moreover, an upregulated miR-100 attributed to poor overall and disease-free survival in T-ALL is involved in reduced lipid accumulation in metabolic syndromes (62) and probably involved in inositol phosphate metabolism in multiple myeloma (38). Similarly, miR-124a and miR-128b were found to be downregulated in T-ALL, and are associated with poor prognosis, and have a role in the inhibition of the Warburg effect and suppression of glucose metabolism, respectively (40, 42). Furthermore, the enhanced miR-221 and miR-222 in T-ALL are not yet known to have any metabolic reprogramming function in cancer, but they are reported to have roles in adipogenesis in obese conditions (45). An underexpressed miR-326 is found to impact key glucose metabolism regulators (47). The significance of miR-708, which is found underexpressed in T-ALL and correlates with poor survival, has not yet been investigated for its metabolic role in cancer, but it has been reported to affect glucose metabolism by influencing insulin secretion (49) and also influence prostaglandin E2 production, affecting tumorigenesis (48). Thus, these miRNAs with diagnostic and prognostic significance expectedly mold the metabolic status of cancer cells by prominently affecting glucose metabolism to support malignant growth (**Figure 2**).

## 3 Peripheral T-cell lymphomas

Unlike T-ALL, which originates from immature thymocytes, PTCL is a malignancy of mature T cells. It is a heterogeneous class of nodal and some extranodal tumors, corresponding to approximately 10%–15% of all lymphoid malignancies. PTCL can be subdivided into three major types: angioimmunoblastic T-cell lymphoma (AITL), anaplastic large-cell lymphoma (ALCL), and peripheral T-cell lymphoma-not otherwise specified (PTCL-NOS) (63). As far as the diagnosis of PTCLs is concerned, conventional diagnostic methodologies have not performed efficiently, and therefore, more than 30% of all PTCL cases remain unclassified and remain at the intersection of various subtypes. Although at the molecular level, these subgroups, despite sharing an overwhelming number of commonly altered pathways and transcriptional signatures, have distinct expression levels revealed by gene expression profiling of patients (64). MicroRNA profiling of PTCL cases revealed specific miRNA signatures based on differential expression of various miRNAs when compared to normal cells. Many miRNA profiling studies, however, have reported differentially expressed miRNAs within various subtypes (65), but their diagnostic accuracies will depend on their practical ability to discriminate the subtypes at the molecular level. A notable miRNA, miR-187 overexpression, is associated with peripheral T-cell lymphoma progression, which is also related to sensitivity to bortezomib (66) and is associated with better survival in other cancers (67). However, its clinical role in PTCL patient outcomes and prognosis remains unclear.

### 3.1 Angioimmunoblastic T-cell lymphoma

AITL is a rare but the second most frequently reported malignant lymphoma of peripheral T cells that originates in mature T lymphocytes. It also accounts for around 1%–2% of all non-Hodgkin lymphoma (NHL) cases. Clinically, AITL cases manifest symptoms such as lymphadenopathy, hepatosplenomegaly, hypergammaglobulinemia, and anemia. Less common symptoms include arthritis, ascites, and some autoimmune characteristics, such as circulating rheumatoid antibodies, antismooth muscle antibodies, and hemolytic anemia (68). Generally, death in AITL patients occurs due to overwhelming immunodeficiency and not due to a serious tumor load (69). AITL pathogenesis and progression are also associated with EBV; in some cases, its onset closely mimics an EBV infection confirmed by EBV-specific antibodies in AITL-positive patient serology (70). miRNA profiling of 30 patients with AITL revealed suppressed expression of four miRNAs, miR-140-3p, let-7g, miR-30b, and miR-664, whereas three miRNAs, miR-146a, miR-193b, and miR-34a, were significantly upregulated (71). Among these, miR-146a and miR-34a influence the expression of the EBV-associated protein LMP1 in an NF-κB-dependent manner, thus helping in tumorigenesis; however, these miRNAs are also overexpressed in B-cell lymphomas (72), indicating their lower specificity for AITLs.

**Figure 3:**
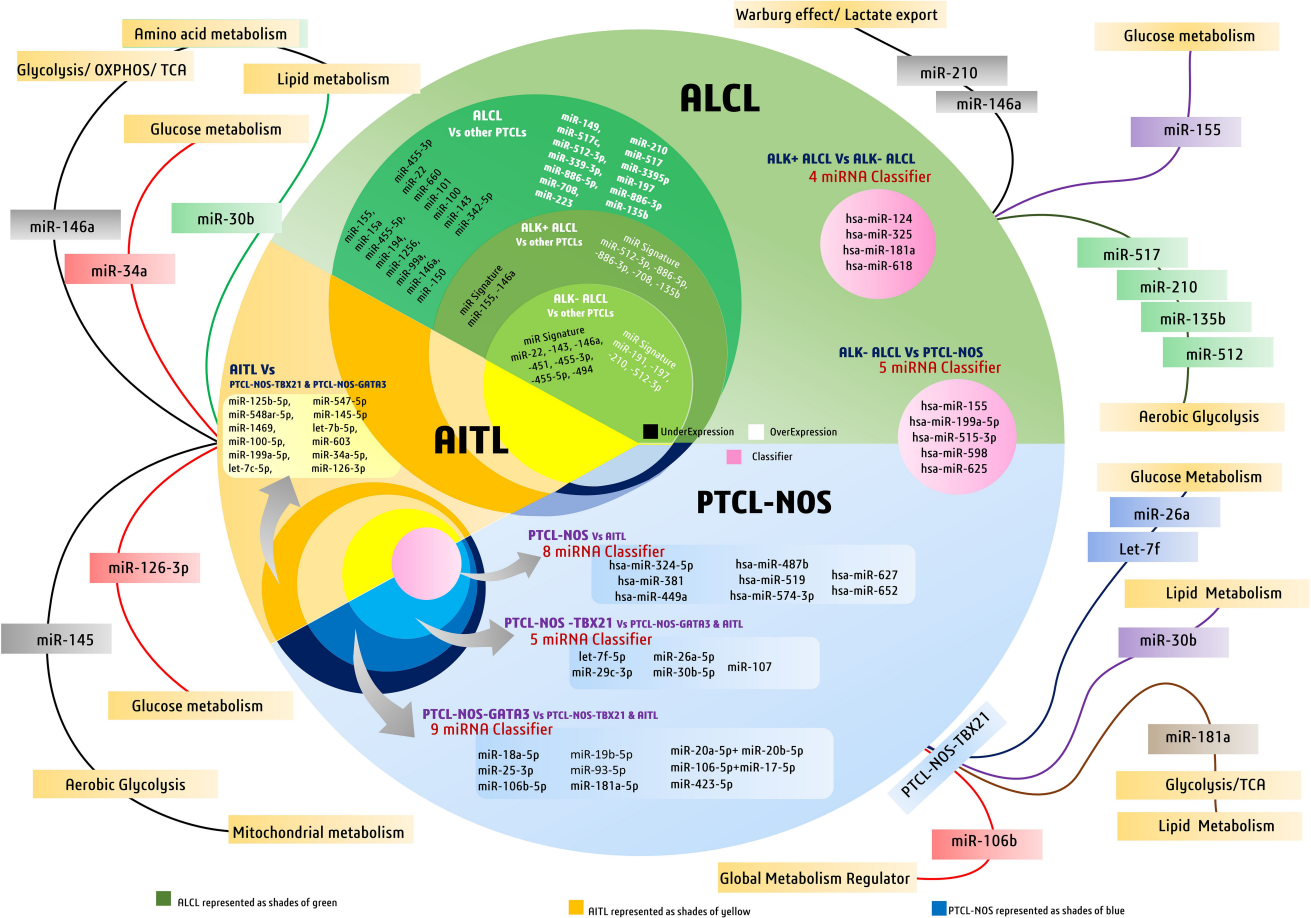
Discriminatory miRNAs and miRNA signatures in major peripheral T-cell lymphoma (PTCL) subtypes like PTCL-NOS, AITL, and ALCL. The figure also highlights major diagnostic miRNAs of PTCLs involved in metabolic regulation in cancers.

Genome-wide miRNA expression profiling of AITL biopsy samples when compared with their polarized CD4+ T-cell counterparts, produced by polarizing them and grouping them based on their cytokine signatures, revealed many overlapping and distinct miRNA signatures. In general, AITL was associated with many differentially expressed miRNAs compared with their normal counterparts or other subtypes of PTCL-NOS, such as the PTCL-GATA3 and PTCL-TBX21 subtypes. These 13 differentially expressed miRNAs (**Table 2**) signify a distinct miRNA signature that distinguishes AITL from other T-cell malignancies. Among these miRNAs, miR-126-3p is important as it regulates angiogenesis (85), maintains endothelial vasculature (86), and has roles in T-cell activation (87) and differentiation (88), all of which are interesting roles in the context of AITL pathobiology. Moreover, two miRNAs, miR-126-3p and miR-145-5p, are important because they show marginal and significant associations with AITL overall survival (OS) (**Figure 3**). Ectopic expression of miR-126-3p in cell line models showed repression of sphingosine-1 phosphate receptor-2 (S1PR2), which has been reported to activate RhoA signaling and is associated with poor prognosis, whereas ectopic expression of miR-145-5p represses ROCK1, which is a downstream target of RhoA. Hence, these miRNAs, along with their association with poor prognosis, are also responsible for regulating Rho-GTPase and inhibiting T-cell migration, as observed in AITL pathobiology (77). These enriched miRNA signatures, when fed into an *in silico* computational annotation tool (89) for pathway analysis, showed the involvement of two major pathways. These enriched miRNAs activated T-cell receptor signaling along with the activation of PI3K-Akt signaling (77). Therefore, these overexpressing miRNAs and their involvement in peculiar AITL pathobiology hold promising potential and call for more focused investigations.

**Table 2:** PTCL subtypes associated with miRNA/miRNA-Classifier carrying clinical/molecular relevance.

PTCL subtype	miRNA	Status	Clinical relevance/molecular relevance	Metabolic role	References
PTCL	miR-187	OE	PTCL progression, sensitive to bortezomib	Decreases glucose-stimulated insulin secretion. Role in cancer metabolism unknown	Locke et al. (73) and Yan et al. (66)
AITL	miR-146a	OE	Target TRAF and IRAK1 and negatively regulate the NF-KB pathway	Global regulator of cancer metabolism	Bogusławska et al. (74) and Reddemann et al. (71)
AITL	miR-34a	OE	Inhibits sirtuin-1 (SIRT1), which stimulates the NF-KB pathway	Negative regulator of LDHA expression and glucose metabolism	Reddemann et al. (71) and Zhang et al. (75)
AITL	miR-30b	UE	Targets Delta-like ligand 4 (DLL4) of the NOTCH signaling pathway	Negatively regulated lipid metabolism	Reddemann et al. (71) and Zhang et al. (76)
AITL	miRNA-126-3p	OE	Poor survival outcome, activates RhoA signaling	Role in glycometabolism in cardiovascular diseases, role in cancer metabolism unknown	Lone et al. (77) and Ma et al. (78)
AITL	miR-145-5p	OE	Poor survival outcome	Negatively regulates aerobic glycolysis and its role in mitochondrial metabolism	Hu et al. (79) and Zhao et al. (80)
AITL vs. PTCL GATA3 and PTCL-TBX21	12 miRNAs: miR-125b-5p, miR-497-5p, miR-548ar-5p, miR-145-5p, miR-1469, let-7b-5p, miR-100-5p, miR-603, miR-199a-5p, miR-34a-5p, let-7c-5p, miR-126-3p	OE	Various T-cell receptor signaling, PI2K-Akt signaling, RhoA pathway	x	Lone et al. (77)
ALCL vs. other PTCLs	13 miR signatures: miR-149, miR-210, miR-517c, miR-517a, miR-512-3p, miR-339-5p, miR-339-3p, miR-197, miR-886-5p, miR-886-3p, miR-708, miR-135b, miR-223	OE	No data	x	Liu et al. (81)
ALCL vs. other PTCLs	15 miR signatures: miR-155, miR-455-3p, miR-15a, miR-22, miR-455-5p, miR-660, miR-194, miR-101, miR-1256, miR-100, miR-99a, miR-143, miR-146a, miR-342-5p, miR-150	UE	No data	x	Liu et al. (81)
ALK+ ALCL	miR-13b	OE	Growth promotion by targeting FOXO1	x	Matsuyama et al. (82)
ALK+ ALCL	miR-29a	UE	Modulates apoptosis by inhibiting MCL-1	x	Desjobert et al. (83)
ALK+ ALCL	11 miRNA signatures: miR-26a, miR-29a, miR-29b, miR29c, miR-30a, miR-101, miR-142-3p, miR-142-5p, miR-145, miR-146a, miR-150, miR-343-3p, and miR-451	UE	No data	x	Liu et al. (81)
ALK+ ALCL vs. other PTCLs	5 miR signatures: miR-512-3p, miR-886-5p, miR-886-3p, miR-708, miR-135b	OE	x	x	Liu et al. (81)
	2 miR signatures: miR-155, miR-146a	UE	x	x	Liu et al. (81)
ALK− ALCL vs. other PTCLs	4 miR signatures: miR-191, miR-197, miR-210, miR-512-3p	OE	x	x	Liu et al. (81)
	7 miR signatures: miR-22, miR-143, miR-146a, miR-451, miR-455-3p, miR-455-5p, miR-494	UE	x	x	Liu et al. (81)
ALK− vs. ALK+	4 miRNA classifier: hsa-miR-124, hsa-miR-325, hsa-miR-181a, hsa-miR-618	Classifier	x	x	Laginestra et al. (63)
PTCL-NOS vs. others	20 miRNA signatures: kshv-miR-K12-12-5p, hsa-miR-1290, hsa-miR-5585-3p, hsa-miR-593-3p, hsa-miR-4763-3p, hsa-miR-3196, hsa-miR-4714-5p, hcmv-miR-UL148D, hsa-miR-4485, hsa-miR-4769-3p, hsv1-miR-H17, hsa-miR-1587, hsa-miR-4674, hsa-miR-3913-5p, hsa-miR-614, hsa-miR-224-3p, hsa-miR-4694-3p, hsa-miR-513c-3p, hsa-miR-4677-3p, hsa-miR-218-2-3p	Classifier	x	x	Lin et al. (84)
PTCL-NOS vs. ALK− ALCL	5 miRNA classifier: hsa-miR-155, hsa-miR-199a-5p, hsa-miR-515-3p, hsa-miR-598, hsa-miR-625	Classifier	x	x	Laginestra et al. (63)
PTCL-NOS vs. AITL	8 miRNA classifier: hsa-miR-324-5p, hsa-miR-381, hsa-miR-449a, hsa-miR-487b, hsa-miR-519, hsa-miR-574-3p, hsa-miR-627, hsa-miR-652	Classifier	x	x	Laginestra et al. (63)
PTCL-NOS TBX21 vs. AITL/PTCL-NOS GATA3	5 miRNAs: let-7f-5p, miR-29c-3p, miR-26a-5p, miR-30b-5p, miR-107	OE	x	x	Lone et al. (77)
PTCL-NOS GATA3 vs. AITL/PTCL-NOS TBX21	9 miRNAs: miR-18a-5p,miR-25-3p, miR-106b-5p, miR-181a-5p, miR-93-5p, miR-20a-5p+ miR-20b-5p, miR-423-5p, miR-19b-5p, miR-106-5p+miR-17-5p	OE	x	x	Lone et al. (77)

### 3.2 Anaplastic large cell lymphoma

ALCL is a malignancy of mature T lymphocytes characterized by anaplastic characteristics, such as horseshoe-shaped nuclei with large pleomorphic lymphoid cells. Molecularly, ALCL is further subdivided into two groups, ALK + ALCL and ALK− ALCL, based on rearrangement (translocation) of the anaplastic lymphoma kinase (ALK) gene (90). Diseases in which this gene is rearranged give rise to the expression of chimeric nucleophosmin (NPM) protein, which causes constitutive ALK expression (91). This translocation and eventual formation of the fusion protein NPM-ALK is the definitive molecular feature of ALK+ ALCL, whereas the other subgroup, that is, ALK− ALCL, is coarsely defined as cases devoid of expressing the fusion protein NPM-ALK. This NPM-ALK fusion protein activates many growth-promoting and anti-apoptotic pathways, such as cJun, cMyc, Jak/STAT, and PI3K-Akt/mTOR (92). In a major study in which miRNA profiling of ALCL cases was performed and compared to miRNA profiles of other PTCL cases, such as AITL and PTCL-NOS, diagnostic miRNA signatures were identified after nullifying miRNAs that were also expressed in stromal cells. This diagnostic miRNA signature differentiates ALCL from other PTCL cases and comprises 13 upregulated miRNAs (**Table 2**), led by the highest expression of miR-149 along with others, and 15 downregulated miRNAs (**Table 2**), led by miR-155. These miRNA signatures do hold promising diagnostic potential owing to their discriminatory nature regarding various molecular and clinical manifestations of PTCLs (81).

#### 3.2.1 ALK+ and ALK− ALCL

ALK+ ALCL has positive expression of the oncogene fusion protein NPM-ALK, which promotes miR-135b expression by activating signal transducer and activator of transcription 3 (STAT3). This miR-135b also targeted FOXO1 and inhibited its growth suppression activities (82). As STAT3 activation is essential for ALK-mediated transformation, its knockdown in ALK cells and forced expression of a certain miRNA cluster (miR-17-92) result in STAT3 rescue, suggesting a positive role of this cluster in STAT3 and ALK-mediated transformation (93). Similar to most ALCL phenotypes that overexpress anti-apoptotic genes like myeloid cell leukemia-1 (MCL1), it has been reported that this overexpression is done by the NPM-ALK fusion oncoprotein by downmodulating another miRNA called miR-29a (83). However, the role of miR-29a in determining clinical outcomes and patient survival remains unclear.

ALK+ cell lines overexpressed six miRNAs (miR-17, miR-20a, miR-20b, miR-93, miR-106a, and miR-886-3p), where miR-886-3p had very significant overexpression (≥16-fold) when compared with ALK− cell lines (90). Studies on ALK+ ALCL cell lines and ALK+ ALCL clinical samples have revealed many deregulated miRNAs in comparison to their healthy counterparts. Of these deregulated miRNAs, the repressed expression of 11 miRNAs has been reported in ALK+ ALCL (**Table 2**) (81).

To differentiate ALCL subtypes from other PTCL subtypes, research suggests unique miRNA signatures associated with both ALK+ and ALK− subtypes. ALK+ ALCL has a significant association with seven miRNAs, of which the expression of five miRNAs was elevated (miR-512-3p, miR-886-5p, miR-886-3p, miR-708, and miR-135b) and the expression of two miRNAs (miR-146a and miR-155) was repressed (**Table 2**) (81). In contrast, ALK− ALCL was found to be associated with 11 miRNAs, of which four were elevated (led by miR-191) and seven had lower expression (led by miR-22) (**Figure 3**) (81). Therefore, these miRNAs can be used as classifiers to diagnose and differentiate ALK subtypes of ALCL from other PTCL subtypes.

### 3.3 PTCL-NOS (not otherwise specified)

PTCL-NOS consists of a group of malignancies that are not similar to the other main PTCL subtypes. Generally, PTCL-NOS is molecularly characterized by an aberrant T-cell immunophenotype, usually with the loss of CD5 and CD7 (94). Similar to many other PTCLs, PTCL-NOS shows molecular expression similarity to activated CD4+ and CD8+ T lymphocytes in *in vitro* studies. In PTCL-NOS, overexpression of miR-187 has been reported to be associated with tumor progression and a poor prognosis (66). In this regard, many studies have attempted to classify PTCL-NOS cases based on their miRNA expression patterns. A study by Laginestra et al. found 158 miRNAs that significantly affect the PTCL-NOS transcriptome compared to normal activated CD8+ and CD4+ T lymphocytes. Of these miRNAs, there was a significant underexpression of miR-132-3p, a miRNA found to be downregulated in many other solid tumors, but in PTCL-NOS cases, it showed differential expression compared to normally activated CD4+ and CD8+ T cells (63). In addition to the above study, another miRNA profiling study reported a miRNA signature of 20 miRNAs (led by kshv-miR-K12-12-5p) in PTCL-NOS that can be used for diagnostic purposes while narrowing down five miRNAs, namely hsa-miR-224-3p, hsa-miR-614, hsa-miR-3196, hsa-miR-1290, and hsa-miR-4485, (**Table 2**), which related to common cancer progression pathways (84). They reported a significant increase in expression of miR-1290 and miR-4485 in PTCL-NOS among all other subtypes (84).

Although miRNA expression analysis has shown a large number of miRNAs expressed in various subtypes of PTCLs, their diagnostic efficacy has not been assessed in previous studies. Laginestra et al. (63) not only studied differentially expressed miRNAs in PTCL subtypes but also narrowed down the huge number of miRNAs to a set of few miRNA classifiers and devised a practical tool for their diagnostic efficiency. According to this study, PTCL-NOS can be differentiated from ALCL/ALK− using a miRNA classifier comprising five miRNAs (led by has-miR-155) (**Table 2**). Similarly, PTCL-NOS can be distinguished from AITL using another miRNA classifier with eight miRNAs (led by has-miR-324-5p) and a four-miRNA classifier (led by hsa-miR-124) (**Table 2**) to differentiate ALK+ and ALK− ALCLs (63). However, a few other studies have subdivided PTCL-NOS into two groups, namely PTCL-NOS-GATA3 and PTCL-NOS-TBX21, based on patient gene expression profiling (GEP). The PTCL-NOS-GATA3 subtype is associated with PI3KP and mTOR upregulation, whereas PTCL-NOS-TBX21 activates the NF-κB pathway. As far as the PTCL-NOS-GATA3 subtype is concerned, it showed nine distinct miRNAs (led by miR-18a-5p), like a signature peculiar to PTCL-NOS-GATA3 (77). PTCL-NOS-TBX21 also showed five distinct miRNA expressions (led by let-7f-5p) when compared against AITL or other PTCL-NOS-GATA3 cases (**Figure 3**) (77). As the incidence of PTCL-NOS is the highest among all PTCLs, an increasing number of clinical studies validating these miRNA classifiers will help to understand and validate their diagnostic accuracies and their effect on survival outcomes.

### 3.4 Metabolic roles of dysregulated miRNAs in PTCLs

As PTCL has several subtypes, upregulation of microRNA miR-187 reported across PTCL subtypes is known to decrease glucose-stimulated insulin secretion (GSIS) in metabolic syndromes; however, its role in cancer metabolic reprogramming is unknown (73). In AITL, the overexpression of miR-146a has a global implication on cancer metabolic reprogramming, starting from glycolysis, oxidative phosphorylation, and TCA, along with amino acid and lipid metabolism (74). The miR-34a, which is also overexpressed in AITL, has a negative role in regulating glucose metabolism by lowering the expression of lactate dehydrogenase A (LDHA) (95), whereas the downregulated miR-30b has a negative role in regulating lipid metabolism as reported in hepatocellular carcinoma (76). Similarly, an overexpressed miR-126-3p, suggesting poor prognosis in AITL, expectedly is a global regulator of angiogenesis, having a role in neovascularization and glycometabolism in cardiovascular diseases (78). This miR-126 is also suggested to have been enriched in microvesicles (96), indicating its prospective role as a noninvasive circulating biomarker potentially useful for clinical management. Another miRNA, miR-145, associated with poor survival in AITL, finds significance in negatively regulating aerobic glycolysis and has also been found to inhibit mitochondrial metabolism in cervical cancer and ovarian cancer (79, 80).

In ALCL, few miRNAs among the 13 found to have diagnostic specificity against other PTCLs have metabolic roles. Among them are the highly upregulated hypoxia-responsive miR-210 and miR-517, which are implicated in facilitating aerobic glycolysis (75, 97), whereas miR-708 may have a role in facilitating cellular metabolism (48). For ALK+ ALCL, the upregulated diagnostic miRNA signature, including miR-512 and miR-135b, is reported to have an inhibitory effect on glycolysis (98, 99), whereas a repressed miR-155 may signify reduced glucose metabolism (100). Another downregulated miR-146a in ALK+ ALCL may signify an increase in the Warburg effect and lactate export (101) to support cancer growth. Like ALK+ ALCL, miR-210 is also upregulated in ALK− ALCL, which has a well-known role in facilitating the Warburg effect by decreasing tri-carboxylic acid (TCA) cycle activity and increasing lactate production (102). Furthermore, in PTCL-NOS-TBX21, miRNAs let-7f and miR-26a were found to be most upregulated and have well-documented roles in modulating metabolism in cancers (103, 104), whereas an upregulated miR-30b is known to negatively regulate lipid metabolism (76). In PTCL-NOS-TBX21, an upregulated miR-106b is one of the global regulators of cancer metabolism (74). In this TBX21 subtype, an upregulated miR-181a is found to reprogram metabolism to influence glucose metabolism (105), lactate production (106), and lipid metabolism (107), and can inhibit IDH1/2 and TCA cycle enzymes (108). Moreover, an upregulated miR-20 is reported to have been involved in increasing fatty acid synthesis in separate studies (109) on metastatic cancers; therefore, its levels in aggressive forms of PTCL-NOS can be of clinical and diagnostic importance (**Figure 3**).

## 4 Extranodal NK/T-cell lymphomas

Another subtype of PTCL, extranodal NK/T-cell lymphomas, should be discussed separately because of their peculiar nature of affecting non-nodal regions. Almost 80% of the reported cases occur around the nose and the region associated with the nasopharynx and oropharynx (110). As far as the incidence of ENKTCL is concerned, it is more commonly diagnosed in men than in women, along with an ethnic bias where it is reported more in Asian, South American, and Central American populations than in Western countries (111). Apart from its apparent clinical course associated with extensive necrosis, ENKTCL is also associated with Epstein–Barr virus (EBV) infection, a peculiar and defining feature of this malignancy. This malignancy is sustained by upregulation of pro-proliferation pathways like JAK/STAT3-related pathways, NF-κB pathways, RUNX3, platelet-derived growth factor (PDGF) pathway, NOTCH1, and aurora kinase pathway, among others (112).

When considering the role of miRNAs in ENKTCL malignancies, earlier studies have shown the diagnostic significance of circulating miR-221, whose upregulation in plasma is so enriched that it can be quantified without total mRNA extraction (113). miR-221 is also upregulated in T-ALL and associated with shorter survival (56). Earlier studies also found downregulation of miR-26a, miR-26b, miR-28-5p, miR-101, and miR-363 in nasal-type ENKTCL when compared to normal NK cells (114). As more than 90% of ENKTCL cases have EBV infection, EBV-encoded miRNAs also play a significant role in driving the transformation of NK/T cells and evading scrutiny by cytotoxic T cells. Among these EBV-encoded miRNAs, BART9 is directly involved in modulating latent membrane protein 1 (LMP1) expressed in almost all EBV-associated lymphomas, providing ENKTCL and its relationship with BART9 with a discriminatory edge compared to other non-EBV-associated lymphomas (115). Other EBV-encoded miRNAs, such as miR-BART20-5p and miR-BART8, are upregulated and involved in inhibiting the IFN-γ STAT1 pathway (116) and evading immune scrutiny, whereas miR-BART9 was found to directly inhibit transcription factor T-bet, resulting in repressed expression of IFN-γ (117). In non-EBV-associated lymphomas, two miRNAs (miR-205 and miR-142-3p) were found to support tumor progression by upregulating the expression of BCL6 and interleukin-A. Interestingly, these two miRNAs were found to be repressed in EBV-associated lymphomas (**Table 3**) (118), indicating their diagnostic role in discriminating between EBV and non-EBV-linked lymphomas. Various other miRNAs such as miR-21, miR-155, miR-221, miR-223, and miR-4943p are overexpressed, and several others such as miR-15a, miR-16, miR-143, miR-146a, miR-150, and miR-205 are found to be underexpressed in independent studies but have less diagnostic potential because of their association with other malignancies (119).

**Table 3:** Important miRNAs in ENKTCL and their molecular targets.

Serial No.	miRNAs	Expression status	EBV association	Linked pathway/target	Reference
1	BART9	Overexpressed	Yes	Regulates LMP1 levels	Ramakrishnan et al. (115)
2	miR− BART8	Overexpressed	Yes	Inhibits IFN-G STAT1 pathway	Huang and Lin (116)
3	miR-BART20-5p	Overexpressed	Yes	Inhibits IFN-G STAT1 pathway, targets PTEN-AKT-mTOR/RICTOR pathway	Huang and Lin (116) and Chen et al. (117)
4	miR-205	Underexpressed	No	Regulates BCL6 expression	Alles et al. (118)

## 5 Cutaneous T-cell lymphomas

Cutaneous T-cell lymphoma is a heterogeneous group of malignancies with different clinical, histological, and prognostic features that are grouped according to their apparent effects on skin and cutaneous tissues. A general feature of all CTCLs is the presence of malignant T cells proliferating in a chronic inflammatory environment that expands to the skin, like the sezary syndrome (SS) and mycosis fungicides (MF), which are aggressive variants of CTCLs and constitute the majority of the cases (120). Past research has successfully identified miRNA signatures that differentiate CTCLs from other similar diseases/malignancies, for example, benign inflammatory dermatosis (121). These specific miRNA signatures may be potent in not only identifying CTCLs from other tumors/lymphomas but can also differentiate between various subtypes of CTCLs such as MF, erythrodermic mycosis fungoides (eMF), SS and others (122, 123).

In CTCLs, miR-155 has been reported to be upregulated and act as an oncogene across studies. A three-miRNA classifier consisting of upregulated miR-155 along with downregulated miR-203 and miR-205 (tumor suppressors) can diagnose CTCLs from other benign skin diseases with more than 90% confidence interval (**Table 4**) (124). MicroRNA profiling revealed significant upregulation of miR-326, miR-663, and miR-711 and downregulation of miR-203, miR-205, and miR-718 in CTCLs. This study identified miR-326, miR-663, and miR-711 as the most induced miRNAs in CTCL cases. Using miR-103 and miR-425-5p expression as a qPCR reference, three miRNAs, including downregulated miR-203 and miR-205 and upregulated miR-326, were used for PCR validation of CTCL cases with enhanced diagnostic capabilities (124) (**Table 4**).

**Table 4:** MicroRNAs for differential diagnosis of various CTCL subtypes.

CTCL subtype	miRNAs	Expression	Significance	Study
CTCLs	miRNA classifier: miR-155, miR-203, miR-205	miR-155 overexpressed, miR-203 and miR-205 repressed	Differentially diagnose CTCLs from other benign inflammatory skin disorders	Ralfkiaer et al. (124)
	miRNA classifier: miR-155, miR-130b, miR-142-3p, miR-200b, and miR-203	miR-200b and miR-203 are repressed rest miRNAs overexpressed	Diagnose CTCLs from benign inflammatory dermatosis (BID)	Shen et al. (125)
	miRNA classifier: miR-106b-5p, miR-148a-3p, miR-338-3p	Differentially expressed (over)	The diagnostic and predictive significance associated with progression from low risk to high risk	Lindahl et al. (126)
Mycosis fungiodes	miRNA classifier: miR-26a, miR-222, miR-181a, miR-146a	Differentially expressed in inflammatory skin diseases, MF plaques, and MF tumors	Differentially expressed in MF plaques, MF tumors, and inflammatory skin disorders	Manso et al. (121)
	let-7a, let-7d, let-7f	Underexpression	Low expression in metastatic MF	Maj et al. (127)
	miR-155, miR-92a, miR-93	Upregulated	Upregulation in tumor MF (miR-93 most upregulated)	van Kester et al. (123)
	miR-93	Downregulation	miR-93 associated with early MF	Talaat et al. (128)
	miR-155 and miR-203/miR-205	Upregulated miR-155 and downregulated miR-203/miR-205	Differentiate CTCLs from other malignancies	Dusílková et al. (129)
	miR-34a, miR-93-5p, miR-181a, miR-181b	Upregulated	Differentiate MF from others	Marosvári et al. (130)
	39 miRNA group: miR-142-3p, miR-150-5p, miR-146b-5p, miR-483-3p, miR-940, miR-766-3p, miR-29a-3p, miR-342-3p, miR-128-3p, miR-484, miR-32-5p, miR-425-5p, miR-424-5p, miR-433-3p, miR-625-3p, miR-181c-5p, miR-29b-3p, miR-138-5p, miR-26a-2-3p, miR-877-5p, miR-517a-3p, miR-542-5p, miR-874-3p, miR-539-5p, miR-34c-5p, miR-29c-3p, miR-185-5p, miR-425-3p, miR-215-5p, miR-503-5p, miR-652-3p, miR-449a, miR-181d-5p, miR-627-5p, miR-135a-5p, miR-642a-5p, miR-454-3p, miR-30a-5p, miR-196a-5p	Differentially expressed	Exclusively expressed in early MF and not in psoriasis	Sørensen et al. (131)
	hsa-miR-423-5p, hsa-miR-361-3p, hsa-miR-652-3p, hsa-miR-484, hsa-miR-766-3p, hsa-miR-142-3p, hsa-miR-142-5p, hsa-miR-21-5p, hsa-miR-146a-5p, hsa-miR-155-5p, hsa-miR-21-3p, hsa-miR-93-5p, hsa-miR-425-5p, hsa-miR-130b-3p, hsa-miR-223-3p, hsa-miR-146b-5p, hsa-miR-185-5p, hsa-miR-150-5p, hsa-miR-342-3p, hsa-miR-345-5p, hsa-miR-22-3p, hsa-miR-15a-5p, hsa-miR-34a-5p, hsa-miR-29c-3p, hsa-miR-29a-3p, hsa-miR-22-5p, hsa-miR-424-5p, hsa-miR-7-5p, hsa-miR-18a-5p, hsa-miR-18b-5p, hsa-miR-128-3p, hsa-miR-352-5p, hsa-miR-454-3p, hsa-miR-1248, hsa-miR-16-1-3p, hsa-miR-181a-3p, hsa-miR-501-5p, hsa-miR-326, hsa-miR-425-3p, hsa-miR-340-5p, hsa-miR-424-3p, hsa-miR-503-5p, hsa-miR-32-5p, hsa-miR-495-3p, hsa-miR-377-3p, hsa-miR-127-5p, hsa-miR-381-3p, hsa-miR-337-5p, hsa-miR-376b-3p, hsa-miR-188-5b, hsa-miR-154-3p, hsa-miR-431-5p, hsa-miR-215-5p, hsa-miR-301a-3p, hsa-miR-450a-5p, hsa-miR-629-5p, hsa-miR-330-3p, hsa-miR-9-3p, hsa-miR-33b-5p, hsa-miR-642a-5p, hsa-miR-138-5p, 34c-5p, hsa-miR-18a-3p, hsa-miR-370-3p, hsa-miR-671-5p, hsa-miR-874-3p, hsa-miR-30b-3p, hsa-miR-584-5p, 31-5p, hsa-miR-31-3p, hsa-miR-663a, hsa-miR-483-3p, hsa-miR-940, hsa-miR-433-3p, hsa-miR-410-3p, hsa-miR-212-3p, hsa-miR-26a-2-3p, hsa-miR-517a-3p, hsa-miR-34b-3p, hsa-miR-625-3p, hsa-miR-487b-3p, hsa-miR-539-5p, hsa-miR-181c-5p, hsa-miR-181d-5p, hsa-miR-522-5p, hsa-miR-373-3p, hsa-miR-193a-3p, hsa-miR-329-3p, hsa-miR-627-5p, hsa-miR-135b-5p, hsa-miR-135a-5p, hsa-miR-346, hsa-miR-449a, hsa-miR-589-5p, hsa-miR-877-5p, hsa-miR-509-3p, hsa-miR-598-3p, hsa-miR-187-3p	Upregulated	Upregulated in early MF plaque lesions vs. healthy controls	Sørensen et al. (131)
	10 miRNAs: hsa-miR-204-5p, hsa-miR-375, hsa-miR-99a-5p, hsa-miR-125b-5p, hsa-miR-30a-3p, hsa-miR-10b-5p, hsa-miR-10a-5p, hsa-miR-338-5p, hsa-miR-149-5p, hsa-miR-196a-5p	Downregulated	Downregulated in early MF plaques lesions vs. healthy controls	Sørensen et al. (131)
	81 miRNAs: hsa-miR-188-5p, hsa-miR-517a-3p, 34b-3p, hsa-miR-16-1-3p, hsa-miR-142-3p, hsa-miR-155-5p, hsa-miR-146a-5p, hsa-miR-21-5p, hsa-miR-15a-5p, hsa-miR-425a-5p, hsa-miR-130b-3p, hsa-miR-21-3p, hsa-miR-18b-5p, hsa-miR-223-3p, hsa-miR-146b-5p, hsa-miR-363-5p, hsa-miR-34a-5p, hsa-miR-29a-3p, hsa-miR-345-5p, hsa-miR-181a-3p, hsa-miR-18a-3p, hsa-miR-484, hsa-miR-150-5p, hsa-miR-343-3p, hsa-miR-766-3p, hsa-miR-326, hsa-miR-7-5p, hsa-miR-1248, hsa-miR-874-3p, hsa-miR-18a-5p, hsa-miR-301a-3p, hsa-miR-501-5p, hsa-miR-187-3p, hsa-miR-433-3p, hsa-miR-424-3p, hsa-miR-625-3p, hsa-miR-30b-3p, hsa-miR-330-3p, hsa-miR-212-3p, hsa-miR-181d-5p, hsa-miR-128-3p, hsa-miR-423-5p, hsa-miR-361-3p, hsa-miR-26a-2-3p, hsa-miR-483-3p, hsa-miR-214-5p, hsa-miR-9-3p, hsa-miR-127-5p, hsa-miR-154-3p, hsa-miR-663a, hsa-miR-381-3p, hsa-miR-31-5p, hsa-miR-31-3p, hsa-miR-495-3p, hsa-miR-940, hsa-miR-181c-5p, hsa-miR-340-5p, hsa-miR-337-5p, hsa-miR-887-3p, hsa-miR-542-5p, hsa-miR-877-5p, hsa-miR-329-3p, hsa-miR-671-5p, hsa-miR-346, hsa-miR-615-3p, hsa-miR-370-3p, hsa-miR-33b-5p,-376b-3p, hsa-miR-193a-3p, hsa-miR-509-3p, hsa-miR-598-3p, hsa-miR-135b-5p, hsa-miR-602, hsa-miR-584-5p, 34c-5p, hsa-miR-654-5p, hsa-miR-22-5p, hsa-miR-629-5p, hsa-miR-539-5p, hsa-miR-373-3p	Upregulated	Upregulated in early MF patch lesions vs. healthy controls	Sørensen et al. (131)
	9 miRNAs: hsa-miR-204-5p, hsa-miR-99a-5p, hsa-miR-30a-5p, hsa-miR-10b-5p, hsa-miR-30a-3p, hsa-miR-375, hsa-miR-338-3p, hsa-miR-10a-5p, hsa-miR-125b-2-3p	Downregulated	Downregulated n early MF patch lesions vs. healthy controls	Sørensen et al. (131)
	13 miRNAs: hsa-miR-139-3p, hsa-miR-199a-5p, hsa-miR-127-3p, hsa-miR-196a-5p, -142-5p, hsa-miR-21-5p, hsa-miR-21-3p, 223-3p, hsa-miR-18a-5p, hsa-miR-22-5p, 322-3p, hsa-miR-32-5p, hsa-miR-766-3p	Differentially expressed	Early MF plaque lesions and patch lesions	Sørensen et al. (131)
LCT-MF	miR-155, miR-223	Overexpressed in FMF	Differentiate FMF from LCT-MF	Marosvári et al. (130)
	miR-181b, miR-326	Overexpressed in LCT-MF	Differentiate FMF from LCT-MF	Marosvári et al. (130)
	miR-21-3p, miR-146a-3p, miR136-5p, miR-889, and miR-539-3p	Overexpressed in LCT-MFs	Differentiates LCT-MFs from non-LCT-MFs	Di Raimondo et al. (132)
	miR-708-5p, miR-5701, and miR-3653	Downregulated in LCT-MFs	Differentiates LCT-MFs from non-LCT-MFs	Di Raimondo et al. (132)
Sezary syndrome (SS-MF)	miR-15a, miR-16, miR-150, miR-191 along with miR-223, and miR-17-5p	Overexpressed in SS-MFs	Differentiates SS-MFs from non-SS-MFs	Ballabio et al. (133)
	miR-155	Overexpression in SS-MFs	Higher expression in SS-MF than in MF	Fava et al. (134)
	miR-22-3p, miR-199a-3p, mi-R-199a-5p, miR-199b-5p	Overexpression	Overexpression in SS-MF and eMF compared to early-stage MF	Rittig et al. (135)
	miR-433-3p, miR-27b-5p, miR-663a, miR-342-3p, miR-484, miR-328-3p, and miR-483-3p	Lower expression	Lower expression in SS-MF and eMF compared to early-stage MF	Rittig et al. (135)

### 5.1 Mycosis fungoides

One of the most prevalent types of CTCL is the indolent form known as MF, which accounts for almost 50% of all CTCL cases (120). An early-stage MF resembles small patches and plaques on the skin that advance to form large multifocal itchy lesions and cutaneous tumors in later stages, which may start disseminating malignant T cells to the surrounding nodes, peripheral blood, and vital organs (136). MFs can also be characterized in many groups based on their different clinical and pathological presentations (for example, folliculotropic MF, erythrodermic MF, unilesional MF, hyperpigmented MF, pagetoid MF, granulomatous MF, granulomatous slack skin MF, and vasculature atropicans MF) (137, 138).

In MF, several miRNAs have been implicated to not only have diagnostic potential but also act as oncogenes and drive the pathogenesis of MF. One of them is miR-155, whose upregulation has been consistently observed in various studies concerning MFs. Considering MF’s resemblance to inflammatory dermatoses, differential expression of microRNAs in both disease types is reportedly apparent (121, 139). A study observed significantly higher upregulation of miR-155, miR-92a, miR-92b, and miR-93 in MFs than in inflammatory dermatoses, among which miR-93 showed the highest upregulation. Many miRNAs, including miR-93, miR-146a, miR-181, miR-203, miR-205, miR-16, miR-342-3p, and miR-146b-5p, can diagnose MFs early and efficiently (121) (140). These miRNAs can also be used for differential diagnosis of MFs. Among these, miR-93 is important, as its levels are suggestive of specific stages of MFs. In advanced MFs, miR-93 is upregulated when compared with inflammatory dermatosis, whereas another study suggested its downregulation in early MFs when compared with normal and eczema cases (128).

Although using single miRNAs, such as miR-155, may not be very specific for MFs, its high expression is also seen in other benign forms, such as inflammatory dermatoses. In another study, a four-miRNA classifier with miR-181a, miR-146a, miR-222, and miR-26a could differentiate MF plaques from inflammatory dermatoses. The same study reported that miR-26a and miR-222 were upregulated in early MFs, whereas miR-146a and miR-181a were upregulated in tumoral MFs (121). A study on miRNA profiling in various stages of MFs, in contrast to atopic dermatoses, identified various deregulated miRNAs. Upregulated miR-155, downregulated miR-203, and miR-205 have been reported to distinguish early MFs against atopic dermatoses. A miRNA classifier consisting of upregulated miR-302, miR-155, miR-150, miR-940, and miR-1913 and downregulated miR-149, miR-141, miR-205, miR-221, miR-203, miR-27b, miR-23b, and miR-let7b is deregulated between MF stage 1 and atopic dermatoses. On the other hand, a miRNA classifier with upregulated miR-181a, miR-93, miR-92a-1, miR-107, and miR-15b and downregulated miR-302c, miR-10a, and miR-31 has been found to distinguish MF stages 1 and 2. Another group of deregulated miRNAs consisting of overexpressed miR-93, miR-451, miR-425, miR-142-5p, and miR-421 and downregulated miR-34b, miR-193b, miR-205, miR-1247, and others can discriminate between MF stages 1 and 3, stage 4, and PTCL-not otherwise specified cases (141).

Early MF lesions manifest as plaques and patches which may appear similar to skin inflammation. Therefore, a more recent observation factored out commonly expressed miRNAs in early MF lesions and skin inflammation (psoriasis) to find 39 miRNAs differentially expressing in early MF (**Table 4**) and 12 miRNAs differentially and exclusively expressing in psoriasis cases, while 70 differentially expressed miRNAs remain common in early MF and psoriasis (131). When healthy controls were compared with early plaque and patch lesions, respectively, many miRNAs were differentially expressed (> 2-fold changes) in these lesions (**Table 4**). Interestingly, upregulation of miR-142-5p, miR-21-3p, and miR-155-5p was recorded in both plaque and patch lesions of early MF. When plaque and patch lesions are considered, they differentially expressed 13 miRNAs (**Table 4**), of which 11 miRNAs led by miR-142, miR-21, and miR-22 were found highly expressed in infiltrated plaque lesions and not in patch lesions. Levels of miR-155 and miR-146 remain similar in both MF lesion types (131).

**Figure 4:**
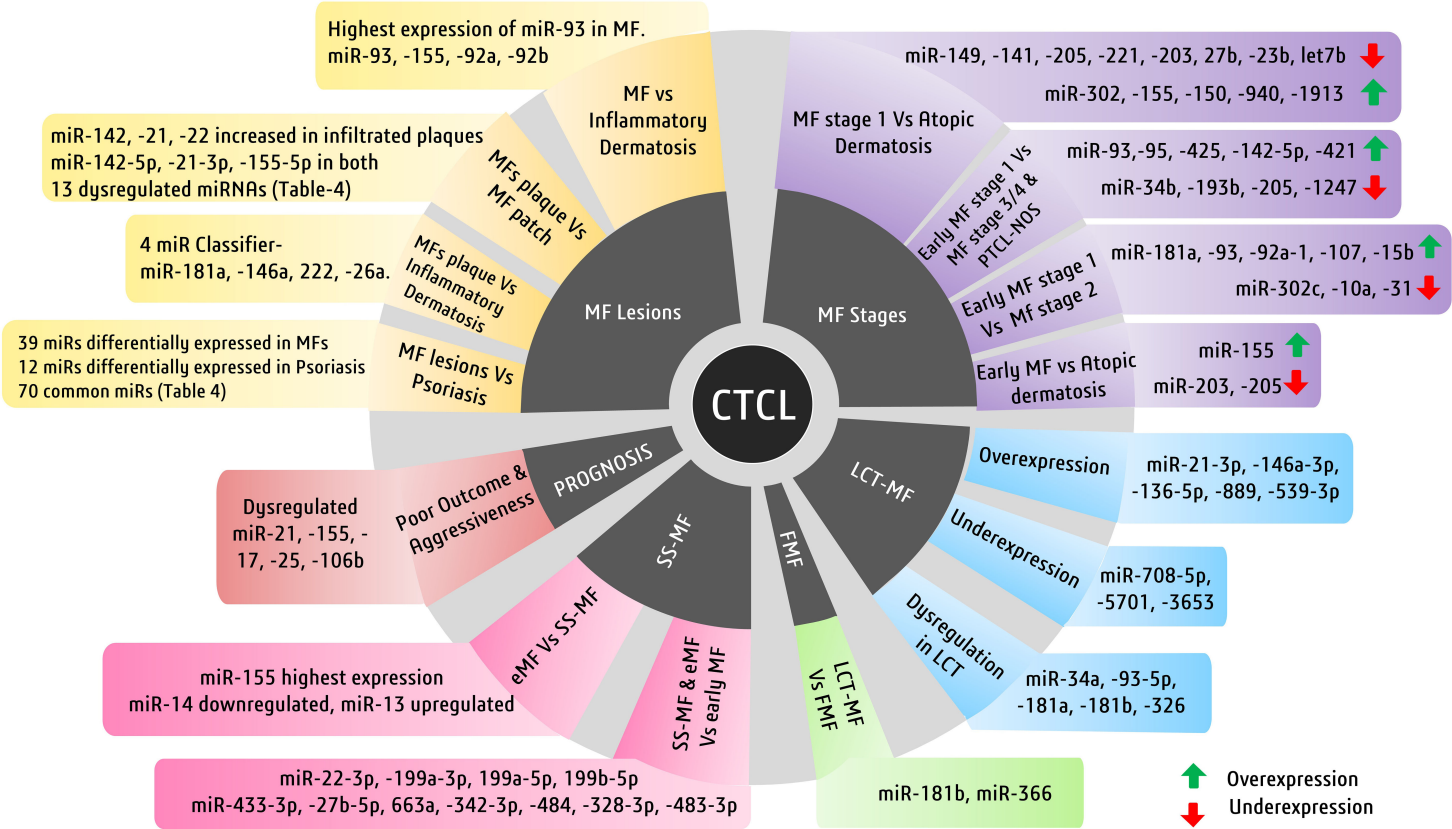
Major cutaneous T-cell lymphoma (CTCL) subtypes and miRNAs of diagnostic and prognostic significance. Illustrated here are the miRNAs involved in mycosis fungiode (MF) diagnosis in a stage- and lesion-specific diagnosis.

Interestingly, few studies detecting miRNAs in plasma found upregulated miR-155 and downregulated miR-203/miR-205 in MF patients with 100% specificity (129). When it comes to predicting clinical outcomes of MF patients, deregulated miR-21, miR-155, miR-17, miR-25, and miR-106b are related to poor outcome and are associated with progressive MF disease (**Figure 4**) (141).

### 5.2 Large cell transformation of mycosis fungoides diagnosis

Another advanced form of MF, called large cell transformation of mycosis fungoides (LCT-MF), is associated with rapid progression of the disease and poor survival outcomes (142). LCT-MFs are characterized by the presence of large T cells that are almost four times bigger than normal T cells and have been reported as an independent prognostic factor for poor survival in MF and SS (143, 144). These malignant transformed T cells (LCT) are often found in advanced stages of MF or SS and very rarely in early stages (145). A study showed significant upregulation of miR-34a, miR-93-5p, miR-181a, miR-181b, and miR-326 in LCT-MF when compared to control cases (130). Another study focusing on the miRNA profile of patients with LCT-MF found the highest upregulation of miRNAs like miR-21-3p, miR-146a-3p, miR136-5p, miR-889, and miR-539-3p, suggesting its role in tumor progression. They also found significant downregulation of miR-708-5p, miR-5701, and miR-3653, suggesting their tumor-suppressive roles (132). These miRNAs in combination could be used to differentially diagnose LCT-MF from non-LCT-MF cases.

### 5.3 Folliculotropic mycosis fungoides

An aggressive subtype of MF known as folliculotropic MF (FMF), which involves deep infiltration of malignant T cells in and around hair follicles of the patient’s body, has a quite similar miRNA profile to LCT-MF with upregulation of miR-34a, miR-93-5p, and miR-181a (130). Both LCT-MF and FMF cases can be distinguished by upregulated miR-181b and miR-326 in LCT-MF and FMF (130). There have been no discriminatory miRNAs discovered to date to diagnose FMF cases positively.

### 5.4 Sezary syndrome

SS is a rare form of CTCL that can be distinguished from other types of lymphomas molecularly by the expression of CD4+ and morphologically by the cerebriform appearance of the nucleus, commonly known as sezary cells. These sezary cells can be found in the peripheral blood, skin, and lymph nodes of the suffering patient. Among all the miRNAs, miR-21 specifically regulates the oncogenic progression in SS by activating the STAT3 signaling pathway. This activation of the signaling pathway is also strongly regulated by IL-21 cytokine. Hence, targeting the miR-21 can provide a new therapeutic approach to SS management (146).

On the other hand, while miR-155 is implicated in several malignancies of T cells and in discriminating various subtypes of CTCLs, miR-155 expression was reported to be higher in SS-MF in comparison to MFs in general (134). Various miRNA expression studies showed elevated levels of miR-15a, miR-16, miR-150, and miR-191 along with miR-223 and miR-17-5p in SS-MF. Although in validation experiments all the above miRNAs failed to predict SS-MF, a distinct level of miR-223 had correctly predicted SS-MF from nonerythrodermic MFs with 90% accuracy (133). Another study focused on early-stage MF, eMF (advanced stage MF), and sezary syndrome, where they found 27 deregulated miRNAs, out of which 14 miRNAs showed lower expression and 13 miRNAs showed enhanced expression in eMF when compared to SS-MF. When compared with early-stage MF, both SS-MF and eMF had overexpressed four miRNAs (miR-22-3p, miR-199a-3p, miR-199a-5p, and miR-199b-5p) and less expression of seven miRNAs (miR-433-3p, miR-27b-5p, miR-663a, miR-342-3p, miR-484, miR-328-3p, and miR-483-3p) (**Figure 4**) (147).

### 5.5 Diagnostic miRNAs of CTCLs and their metabolic significance

In CTCLs, miR-155 is found upregulated irrespective of its subtype but is interestingly found downregulated in PTCLs, where it has a positive role in regulating and driving glucose metabolism (100, 148, 149). Its increased expression in CTCL subtypes may signify a significant role of glucose metabolism in the pathogenesis of CTCLs. The miR-326, which is upregulated in CTCLs in general more so in MFs, along with its FMF variant, is underexpressed in T-ALL. It is known to modulate glucose metabolism by influencing many key regulators (47). Moreover, upregulated miR-146a in all CTCL subtypes and AITL are well known as global regulators of cancer metabolism, influencing glycolysis and TCA cycle activity (74). Similarly, miR-181 is found to regulate glucose and lipid metabolism is upregulated in PTCL-NOS-TBX21 and CTCL subtypes more prominently in mycosis fungiodes and also in T-ALL (77, 105–107, 121). This indicates a similar contribution of miRNA regulators of glucose metabolism to the metabolic landscape of these T-cell malignancies and, hence, can suggest interesting future studies in the discovery of more accurate diagnostic miRNAs.

## 6 Applicability of miRNA-based diagnosis and new avenues

miRNAs for diagnosis have been of research interest in cancers for a long time now. Its application in the diagnosis of T-cell malignancies is, however, of more importance given the wide range of pathobiological, clinical, and cytogenetic variations in its malignant subtypes (150). To avoid inaccurate clinical judgment (151), it is essential to devise new methods of diagnosis that cannot only capture specific tumor markers but also quantify molecular variations among overlapping clinical features. miRNA profiling can be employed to look for miRNA classifiers for specific patterns in T-cell malignancy subtypes (152). For instance, in AITL biology, miR-146a and miR-34a are associated with EBV-linked AITL cases; therefore, their upregulation can be used to establish EBV-associated AITL cases and rule out non-EBV cases. Similarly, miR-30b is significantly associated with angiogenesis and can be used for the differential diagnosis of AITL from others. Moreover, subtypes like extranodal NK/T-cell lymphomas (ENKTCL) overexpresses EBV-associated miRNAs like miR-BART8, miR-BART9, and miR-BART20-5p, along with non-EBV-associated miRNAs found to be over/underexpressed (116). These miRNAs, in combination, can be effectively used to differentially diagnose and discriminate ENKTCL lymphomas from other subtypes.

Circulating miRNAs have attracted a great deal of interest as potential diagnostic biomarkers because of their stability and detectability. miRNAs derived from extracellular vesicles (EVs) (54) and exosomes (153) may reflect a miRNA signature that may resemble a very early disease condition (154). For instance, miR-126 overexpressed in AITL signifies poor prognosis, and its enrichment in the exosome is found to distinguish early and advanced non-small cell lung cancer (NSCLC), while it is downregulated for advanced NSCLC (155). This miR-126 can be investigated in AITL for its possible correlation with the stage and aggressiveness of AITL and whether its exosomal enrichment in body fluids can be used as a noninvasive tool to monitor disease and suggest better therapies. Similarly, in ALCL, an upregulated miR-223 can have a potential role in inducing chemoresistance, whose expression levels can be quantified in exosomes to potentially diagnose and use for disease surveillance (156). Studies also indicate that an upregulated miR-197 in ALK− ALCL can be investigated for its enrichment in exosomes as it has been implicated in metastatic promotion in lung cancer (157). The above insights project miRNAs and their profiling as powerful tools to be potentially used for differential diagnosis of malignancies with overlapping clinical manifestations.

More recently, liquid biopsies, which include examining circulating miRNAs in bodily fluids, have presented a simple, noninvasive method for early and efficient diagnosis along with disease tracking and surveillance capacities (158). However, assessing tumor-specific miRNA expression by liquid biopsy or miRNA enrichment in EVs or exosomes requires many clinical-based studies to establish their clinical applicability. Currently, the applicability of liquid biopsies in clinical settings is debatable (159). On the one hand, the method provides a noninvasive and easily accessible option that incurs less discomfort and risk to the patient than standard tissue biopsies, evaluation of biomarkers derived from it, remains unstandardized (160). The task of standardizing miRNA profiling data analysis is ongoing as platforms and procedures used by various laboratories differ, causing results to fluctuate and making it difficult to reproduce and compare findings (160). A liquid biopsy also gives clinicians and scientists an upper hand in the early detection of tumors, along with its promising potential to record the molecular heterogeneity of tumors, thus providing scope for precise diagnosis and personalized treatment strategies. However, molecular and genetic complexities of T-cell malignancies can influence miRNA profiles depending on the tumor microenvironment, disease stage, individual patient characteristics, or the presence of any additional disease (161). Moreover, circulating miRNA levels can be affected by a number of preanalytical factors, including sample type, storage conditions, and RNA separation techniques. The processing of samples and the selection of the sample type (serum, plasma, or other biofluids) can affect the stability and production of miRNA (162). For trustworthy diagnostic outcomes and to reduce technical variability, these preanalytical factors must be standardized. The clinical relevance, sensitivity, specificity, and positive predictive value of diagnostic tests must be demonstrated through thorough validation and clinical investigations, just like with any other diagnostic test (163). Additionally, in order to authorize miRNA-based diagnostic tests for clinical use, regulatory authorities demand strong proof to avoid faulty clinical judgments while in use (164).

Tumor-associated miRNAs can be detected and quantified in body fluids such as serum (165) or plasma (166) using techniques such as reverse transcriptase PCR (167), microarray (124), and next-generation sequencing (NGS) that were traditionally used for miRNA profiling studies. Quantitative PCR and microarray analysis are two miRNA detection methods that have limitations in terms of sensitivity and specificity. Results that are falsely positive or falsely negative can be recorded, which can reduce the precision of miRNA-based diagnosis. More reliable and powerful detection techniques such as droplet digital PCR (ddPCR) promise increased sensitivity, absolute quantification and precision, multiplexing capabilities, and resistance to PCR inhibitors, and their ability to detect rare variants, which can lead to better-standardized results than traditionally used methods (168).

In malignancies or otherwise, miRNAs were never singularly used for diagnosis, as there are overlapping miRNAs in various malignant disease phenotypes. Therefore, miRNA classifiers and panels encompassing a wide range of over- and underexpressing miRNAs are fit to be used for diagnostic applications. In this regard, many researchers have already devised miRNA classifiers whose diagnostic applicability has been validated in clinical settings (140, 169, 170). However, research is still underway to identify and validate particular miRNAs linked to better prognosis and accurate diagnosis. In this regard, miRNAs having a metabolic impact on tumors may be investigated for their promising diagnostic potential. Many studies have identified miRNA species that have regulatory roles in reprogramming cell metabolism to support or inhibit cancer progression. Such studies often develop an overexpression system using many strategies, such as transfecting cells with miRNA-viral construct or miRNA mimics to transiently overexpress or inhibit a particular miRNA in order to study their functional roles. A brief summary of the methodological strategies and outcomes of such studies is featured in **Supplementary Table 1**. Future investigations exploring such metabolic miRNAs for their diagnostic potential along with the metabolic status of patients could be potentially utilized for more accurate subtyping and subgrouping of diseases and leading precision medicine efforts.

## 7 Conclusion

Various malignancies of the T-cell display overlapping physical symptoms despite each one having different molecular and disease progression profiles. Even though imaging techniques such as PET and CT scans can efficiently diagnose the location, stage, and spread of cancer, they cannot suggest valuable information regarding the best therapeutic choice with minimum toxicity and maximum survival. However, investigating disease-specific molecular biomarkers in tumor tissues or in circulating fluids is a promising avenue of molecular diagnostics and can reduce cancer mortality by suggesting the best therapeutic choices for better survival outcomes. Studies capturing miRNA signatures and unique miRNAs in miRNAome need more variety in terms of patient diversity in addition to the range of miRNAs used for screening. miRNAs involved in regulating diverse signaling pathways can be included in the panel of miRNAs for better diagnosis and categorization of malignant T-cell subtypes. Therefore, more such studies identifying and evaluating tissue or serum miRNAs derived from EVs or exosomes will help identify specific signature miRNAs for various other clinical outcomes and may lead to early and efficient diagnosis and therapy of this diverse disease group. Future studies can explore miRNAs along with their metabolic impact to devise more precise miRNA panels for differential diagnostic purposes.

## Author contributions

Corresponding and lead authors from Guru Ghasidas Vishwavidyalaya wrote and edited the manuscript. All authors contributed intellectual contributions to designing, editing, and proofreading the manuscript. All authors contributed to the article and approved the submitted version.
