# Supplementary material for: Diagnostic significance of dysregulated miRNAs in T-cell malignancies and their metabolic roles
Source: Front Oncol. 2023 Aug 10;13:1230273. doi: 10.3389/fonc.2023.1230273 (PMC10448964; doi:10.3389/fonc.2023.1230273)
Supplement: Supplementary file 1 [file Table_1.pdf]

Supplementary Table 1
Studies concerning metabolic impact of diagnostic miRNAs of T-cell malignancies with brief methodology

miRNA	Expression status in T-cell malignancy	Expression in study	Methodology used	Metabolic impact	Consequence	Reference study
miR-16-5p	Upregulated (T-ALL)	Overexpressed in *in vitro* cell culture system	Measuring extracellular acidification rate (ECAR, a measure of aerobic glycolysis), oxygen consumption rate (OCR), lactate production and colorimetric ATP assays	Decrease glucose consumption, Decrease lactate production rate, Decreased ATP levels	Sensitize resistant cells to Dox treatment Increases Radiosensitivity	(Zhao et al., 2020) (F. Wang et al., 2019)
let-7e	Downregulated (T-ALL)	Overexpressed in cell lines	Measuring Oxygen concentrations, lactate concentration (L-lactate) and Oil Red O staining	Inhibits Oxidative phosphorylation, adipogenesis and reduced lactate production	Transcriptionally regulates mitochondrial genome (ND4 gene) and rewires metabolism	(Sharma et al., 2021)
miR-17-92	Upregulated (T-ALL)	Overexpressed in cell lines	Measuring aerobic glycolysis (ECAR), Oxygen consumption rate (OCR), lactate quantification, measuring glutamine consumption and ammonia production	Increased aerobic glycolysis, glucose uptake and lactate production, increased glutaminolysis	miR-17-92 co-operates with Myc to increase tumor aggressiveness	(Izreig et al., 2016)
miR-99a-5p	Upregulated (T-ALL)	Overexpressed in cell lines and patient tissues	RT-PCR and western analysis for expressional analysis followed by colorimetric assays for glucose uptake and lactate production, ECAR assay for aerobic glycolysis assessment,	Decreased glucose consumption and lactate production	Overexpression of miR-99a-5p induced apoptosis and inhibited glycolysis	(G. Wang et al., 2022)
miR-125b	Upregulated (T-ALL)	Overexpressed in cell lines	Expression study by RT-PCR. Overexprression in	Supressed glucose	MiR-125b inhibited HK2 involved in	(Hui et al., 2018)

Supplementary Table 1
Studies concerning metabolic impact of diagnostic miRNAs of T-cell malignancies with brief methodology

		and assessed in patient tissue samples	cell line followed by quantification of glucose, lactate and apoptosis	consumption and lactate production	glycolytic regulation and tumor progression	
miR-124	Downregulated (T-ALL)	Overexpressed in cell lines and use of locked nucleic acid (LNAs) for inhibition	Impact of glycolysis assessed by inhibiting glycolysis by varied concentrations of 5-TG (5-thioglucose) and evaluating rate of conversion of labelled glucose. Lactate quantification by colorimetric assay and evaluating cellular oxygen consumption rate	Decrease in glycolytic rate, Increase in Oxygen consumption,	Inhibited Warburg effect and inhibiting cancer growth.	(Sun et al., 2012)
miR-326	Downregulated (T-ALL)	Ectopic expression in cells	Evaluation of apoptosis (caspase3/7 assay) and ATP levels upon ectopic expression was noted.	Induction of apoptosis and reduction in metabolic activity	miR-326 targets PKM2 to drive metabolic change in cells	(Kefas et al., 2010)
miR-146a	Upregulated (AITL)	Transient overexpression in primary tumor cells	Transfecting primary cell with miRNA mimics and evaluating mRNA and protein expression of metabolic followed by metabolite analysis using gas chromatography mass spectrometry.	Alteration in TCA cycle genes and upregulation of pentose phosphate pathway genes	Stimulated proliferation of renal cancer calls	(Bogusławska et al., 2019)
miR-34a	Upregulated (AITL)	Transient overexpression using miRNA	Transfection of cells with miRNA mimics followed by evaluation of key metabolic genes and	Reduced glucose uptake<br>Reduced lactate production	MiR-34a targets LDHA and modulates glycolytic pathway to promote	(R. Zhang et al., 2016)

Supplementary Table 1
Studies concerning metabolic impact of diagnostic miRNAs of T-cell malignancies with brief methodology

		mimics or inhibitors	proteins using RT-PCR and Western blot analysis. Glucose and lactate quantification using colorimetric/fluorometric assay kits		tumor proliferation and invasion.	
miR-30b-5p	Downregulated (AITL)	Overexpression and inhibition system using lentiviral constructs	Monoclonal cell line establishment using lentiviral miRNA construct followed by free fatty acid treatment and evaluation of genes and proteins related to lipid metabolism by RT-PCR, western blot, triglyceride levels and Oil Red O staining.	Decrease in size and number of intracellular lipid droplets and triglycerides. Overexpressing cells showed increased and decreased expression of fatty acid oxidation and lipid synthesis related genes.	miR-30b-5p inhibit fat deposition and modulates lipid metabolism.	(Q. Zhang et al., 2020)
miR-145	Overexpressed (AITL)	Transient expression or knockdown using miRNA mimics or inhibitors	Evaluation of metabolism related genes by Q-PCR in overexpressing cells followed by dual-luciferase assay and chromatic-immunoprecipitation to predict their targets	miR-145 negatively associated with genes involved in metabolic reprogramming.	miR-145 influences aerobic glucolysis though MYC.	(Hu et al., 2023)
miR-210-3p	Upregulated (ALCL)	Transient overexpression in cells	Evaluation of metabolism realted genes and proteins by qPCR and western	Enhanced glucose consumption,	miR-210-3p contributes to warburg effect by	(Du et al., 2020)

Supplementary Table 1
Studies concerning metabolic impact of diagnostic miRNAs of T-cell malignancies with brief methodology

			blotting followed by glucose lactate assay	lactate production	inducing aerobic glycolysis.	
let-7f	Upregulated (PTCL-NOS-TBX21)	Exogenous expression using a transgene	Intracellular glucose uptake assay and lactate assay. Metabolite profiling using Liquid chromatography mass spectrometry	Decreased glucose uptake and lactate production. Reduced production of many metabolites in threonine catabolism pathway	Supresses Warburg effect and growth in cancer cells	(Ma et al., 2014; Shyh-Chang et al., 2013)
miR-181a	Upregulated (PTCL-NOS-TBX21)	Molecular study of cells and patient derived mitochondrial miRNA	Purification of mitomiR-181a-5p followed by evaluation of metabolism related gene and protein expression studies using RT-PCR/qPCR, western blotting, miRNA/mRNA immunoprecipitation assays and immunohistochemistry assays	Reduced mitochondrial cytochrome B, mitochondrial CO2 and mitochondrial membrane potential	miR-181a overexpression resulted in reprogramming of glucose metabolism enabling proliferation	(Zhuang et al., 2020)

Supplementary Table 1
Studies concerning metabolic impact of diagnostic miRNAs of T-cell malignancies with brief methodology

miR-155	Upregulated (CTCLs) Downregulated (PTCLs)	miR-155 deficient mouse models and cells	Use of miR-155 deficient *in vitro* and *in vivo* systems and evaluation of metabolic gene and protein expression by PCR and western blotting. Target prediction done by luciferase reporter assays. OXPHOS measurements and metabolite profiling by LC-MS	Activation of glucose metabolism by increased glucose uptake and lactate production	miR-155 is a key regulator of glucose metabolism supporting tumor growth	(Kim et al., 2018)
